# Flexible Sensory Systems: Structural Approaches

**DOI:** 10.3390/polym14061232

**Published:** 2022-03-18

**Authors:** Chan Park, Byeongjun Lee, Jungmin Kim, Haran Lee, Jeongbeom Kang, Jongwon Yoon, Jonghyeon Ban, Chiwon Song, Seong J. Cho

**Affiliations:** Department of Mechanical Engineering, Chungnam National University (CNU), 99 Daehak-ro, Yuseong-gu, Daejeon 305-764, Korea; cksdl4608@naver.com (C.P.); michael489@naver.com (B.L.); kjm5301@naver.com (J.K.); ktten55@naver.com (H.L.); wjdqjarkd@naver.com (J.K.); jongwon3498@naver.com (J.Y.); haze1013@naver.com (J.B.); song7367@naver.com (C.S.)

**Keywords:** soft electronics, soft electronic design, stretchable electronics

## Abstract

Biology is characterized by smooth, elastic, and nonplanar surfaces; as a consequence, soft electronics that enable interfacing with nonplanar surfaces allow applications that could not be achieved with the rigid and integrated circuits that exist today. Here, we review the latest examples of technologies and methods that can replace elasticity through a structural approach; these approaches can modify mechanical properties, thereby improving performance, while maintaining the existing material integrity. Furthermore, an overview of the recent progress in wave/wrinkle, stretchable interconnect, origami/kirigami, crack, nano/micro, and textile structures is provided. Finally, potential applications and expected developments in soft electronics are discussed.

## 1. Introduction

**Figure 1:**
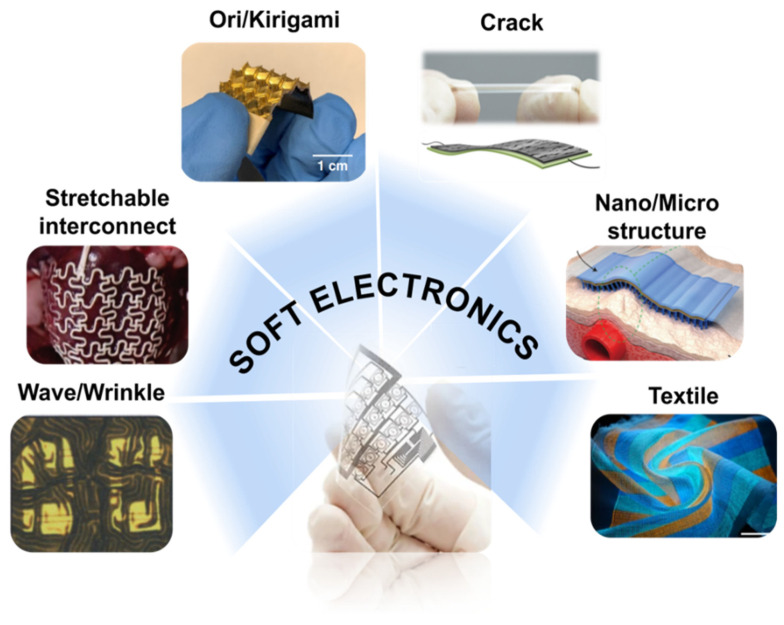
Review summary. Structural design, including wave/wrinkle, stretchable interconnect, origami/kirigami, crack, nano–/microstructure, and textile [13,14,15,16,17,18].

Generally, biology is characterized by smooth, elastic, and nonplanar surfaces [1,2,3,4,5,6]. Therefore, soft electronics that enable interfacing with nonplanar surfaces will allow applications that cannot be achieved with the rigid planar integrated circuits that exist today [7,8,9,10,11,12]. For example, they range from diagnostic tools that attach to the skin for measuring signals, to sensors that accomplish various functions inside the human body [7,8,9,10,11,12]. Some recently published images representing a diverse range of these flexible devices are depicted in Figure 1 [13,14,15,16,17,18].

Owing to the vast range of uses of flexible electronic devices, flexibility requirements are very diverse; flexible devices must be capable of undergoing deformation, and simultaneously, functional properties and electronic performance parameters must be unaffected by the straining process. For example, the electrical resistance of a flexible electrode should be kept at a constant low value from low to high deformation [19,20,21]. Similarly, in the case of flexible solar cells and piezoelectric devices, they should exhibit high efficiencies within acceptable deformation [22,23,24,25,26].

Several advantages are associated with soft electronics, which enable interfacing with nonplanar surfaces. First, the intimate contact between the device and the nonplanar object minimizes noise; this contact increases the effective contact area without any gap between the surfaces. Therefore, signal noise is low, and standard data can be obtained. The contact area is reduced with traditional rigid electronics, potentially introducing noise and artifacts, which compromise signal quality. Second, flexible electronic devices have flexible mechanical properties, thus representing a key technology enabling continuous health management, by minimizing irritation to the skin of the human body. Flexible sensing technology has shown a huge application value in motion monitoring and physiological signal detection. In addition, flexible electronics show good performance in applications for human motion monitoring, such as limb flexion and rotation, as well as muscle strengthening. Finally, flexible, stretchable, and foldable devices have many applications in the internet of things and for monitoring technology. Improving the measurement range is important for many applications: in particular, it can show excellent performance in condition monitoring applications, such as for folding and bending objects.

Recently, various structural approaches have been undertaken to implement soft electronics; these approaches can modify mechanical properties, improving performance, while maintaining the existing material integrity. This paper applies this method to review the latest technologies and methods that can replace elasticity through a structural approach. We demonstrate the many technological advances that would occur if the existing stretchable materials were replaced with nonstretchable materials, through structural design. Furthermore, an overview of recent progress in wave/wrinkle structure, stretchable interconnects, origami/kirigami, crack, nano/microstructure, and textile structures is provided. Finally, potential applications and expected developments of soft electronics are discussed.

## 2. Structural Designs for Flexible Sensory Systems

### 2.1. Wave/Wrinkle Structure

**Figure 2:**
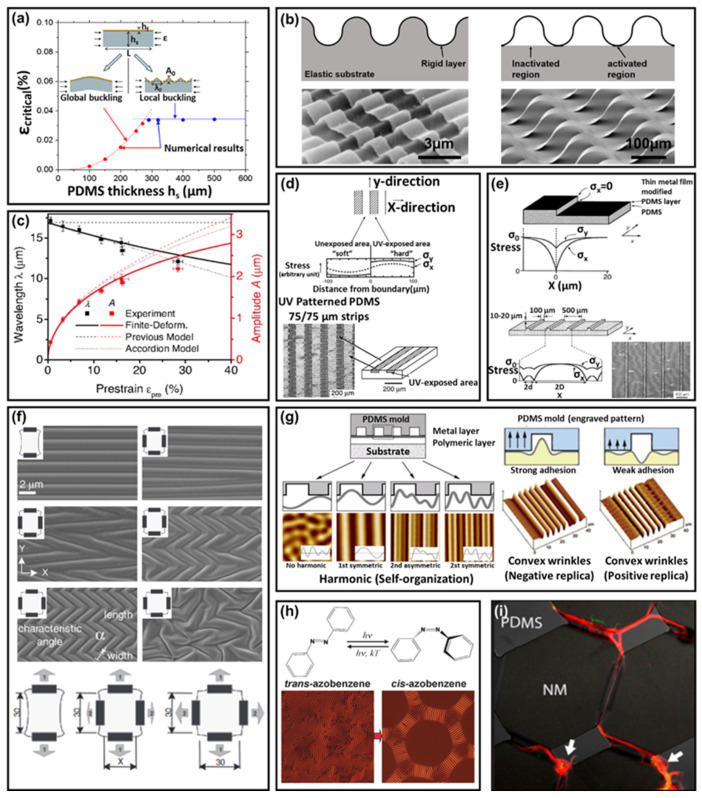
(**a**) Schematic view of buckling mode: global and local buckling of PDMS substrate and Si ribbon bilayer [31]. (**b**) Schematic view of bonded (**left**) and controlled delamination film (**right**) wrinkle structures and SEM micrograph of a natural rubber/Au bilayer (**left**), as well as SEM micrograph of a PDMS substrate/GaAs and Si ribbon (**right**) [28,32]. (**c**) Graph of wrinkle structure wavelength, amplitude of theoretical and experimental value as a function of pre–strain, and wrinkle structure fabricated with Si (100 nm) ribbon on PDMS substrate. As shown, graph small deformation theory is accurate according to an experimental value at below 5% strain, and finite deformation theory is accurate according to an experimental value at above 5% strain [33]. (**d**) Schematic view of ordered wrinkle structure using prepatterning and compressive stress distribution of direction (x,y) [34]. (**e**) Schematic view of ordered wrinkle structures using relief structures that control stress distribution and stress distribution (σ_x_, σ_y_) graph as function of x [35]. (**f**) Schematic view of ordered wrinkle structures using pre–strain that controls stress distribution through adjusting stretch releasing direction and sequence [36]. (**g**) Schematic view of ordered wrinkle structures using mold patterning [37,38,39]. (**h**) Schematic view of ordered wrinkle structures using a photo–softening effect that changes the Young’s modulus and stress distribution through photoisomerization [40,41]. (**i**) Microscope images of wrinkle structure application as a topographic guidance of neural growth [42].

The wave/wrinkle pattern is generated by buckling in a bilayer system with relatively different mechanical and geometrical characteristics [27,28,29,30], determined by the critical strain, which is affected by bilayer material properties and geometric characteristics. As shown in Figure 2a, there are two buckling modes associated with bilayer systems: global and local buckling [31].

Furthermore, wrinkle/wave patterns generated by local buckling have a controllable micro/nanostructure, generally applied to soft electronics, biology, and other fields; thus, this chapter focuses on local buckling wave/wrinkle structures.

In a bilayer system, when an expanded elastic substrate returns to the original volume, compressive stress is generated in the rigid layer during the release process; these kinds of stresses generate mechanical instability, which is relaxed by out–of–plane deformations, which generate a wave/wrinkle pattern; thus, reaching a new equilibrium [27,28].

Wave/wrinkle structures can be classified into bonded, and controlled delamination film (rigid layer), according to the bonding method: elastic layer substrates in the first case, and rigid layer film in the latter. In the case of bonded film wrinkle structures, a rigid layer is completely bonded on an elastic substrate, and the elastic substrate surface deforms to generate wave/wrinkle structures with rigid layers (Figure 2b, left) [28]. In the case of controlled delamination wrinkle structures, part of the rigid layer is strongly bonded to the elastic substrate, due to the patterned surface activation (Figure 2b, right) [32].

#### 2.1.1. Wave/Wrinkle Generation

In the case of bonded film wrinkle structures in a bilayer system, the rigid layer attached to the surface of an elastic substrate deforms to form a wrinkled structure (Figure 2b, left). Both the period and amplitude are predicted by minimizing the total elastic energy at small deformation (small deformation theory); this theory affirms that amplitudes and periods are affected by pre–strain, geometrical characteristics, and mechanical properties [28,29,33]. However, as the pre–strain increases, experimental values differ from the small deformation theory, which assumes a small deformation approximation and a linear stress–strain behavior (as shown in Figure 2c, this theory does not apply to experimental values >5%). On the contrary, large strain theory (finite strain theory), which assumes a nonlinear stress–strain behavior (geometrical nonlinearity) at finite deformation (large deformation) of bilayer systems, can predict theoretical values close to the experimental values at large deformations (as shown in Figure 2c, ~>5%) [30,33,43].

A controlled delaminated film wrinkle structure is a bilayer system, in which part of the rigid layer is strongly bonded to the flexible substrate, due to patterned surface activation, while the nonactivated part is delaminated to form a wrinkled structure (Figure 2b, right) [44,45]. A controlled delamination wrinkle is able to generate the following process: a patterned mask is attached to the expanded elastic substrate, and a patterned surface activation region is generated through oxygen plasma treatment or ultraviolet/ozone(UVO) irradiation. After attaching a rigid layer on the elastic substrate, the elastic substrate relieves volume deformation; as a result, the volume of the elastic substrate shrinks to the original, and compressive stresses occur in the rigid layer. This stress is relaxed by causing delamination between the rigid film and the substrate in the inactivated region, to form a wrinkle. As a result of the delamination wrinkle structures, the bilayer system reaches a new equilibrium state [46]. Furthermore, the theoretical amplitude and period values of controlled delamination wrinkles are affected by the inactivated and activated regions and pre–strain [46,47].

In the wrinkle structures described above, the wrinkle amplitude and period are changed by the applied strain [33]. If it reaches a value equal to ε_p_ + ε_c_ (ε_p_ is pre–strain and ε_c_ is critical strain), the amplitude becomes 0. If the applied strain is increased more than ε_p_ + ε_c_, the deformation is applied directly to the rigid layer. If the applied strain reaches a value equal to ε_p_ + ε_c_ + ε_f_ (ε_f_ is the fracture strain of rigid layer), the rigid layer is fractured. If a compressive strain is applied, fractures occur when the peak strain reaches the fracture strain. Under tensions and compressions, the applied strain can exhibit stretchability and compressibility. As the two values are opposite, depending on the value of the pre–strain, it is possible to design a structure with appropriate stretchability and compressibility by controlling pre–strain [30].

Materials for elastic substrates that can be used for the fabrication of wrinkled structures in bilayer systems include polydimethylsiloxane [48], azo–containing poly (PDO3) [40], and liquid crystal polymer (reactive mesogen) [49]. Regarding elastic substrate expansion, mechanical stretching [48,50], thermal expansion [35,51,52], and solvent swelling [53] can be used. Materials for the rigid layers used in bilayer systems include Au/Pd (Gold and palladium) [54,55], CNT [56], GaAs [57], grapheme [58], SiNMs [59], SiNWs [60], PZT [61], oxygen–plasma–treated [62] and ultraviolet/ozone (UVO)–oxidated elastic substrate surfaces [63], and ZnO [64]. Rigid layer materials are deposited onto an elastic substrate, to form a bilayer using sputtering [54], transfer [60], electroless plating [65], chemical vapor deposition [35], surface cross linking through plasma treatment [52], or UVO irradiation [66,67].

Controllable wrinkle structures with stretchability and compressibility have been applied to various fields (e.g., flexible electronic devices sensor, circuit, biology, etc.). A strain sensor with a wave/wrinkle structure has been reported as an example for applying controllable stretchability and compressibility to detect motion monitoring. The unstructured sensor has a lower strain range (8%), whereas the wrinkle structure strain sensor, generated by pre–strain, improved the strain range (12–65%). Sensors with improved stretchability have been applied to humans and robots, to detect various movements, such as the wrist, leg muscles, and facial expressions [68]. As another example, the wave/wrinkle structure is used as electronics path that is applicable to flexible electronics device. These wrinkle paths are made up of GaAs and Si and have up to ~100% stretchability, ~25% compressibility, and bendability, with a radius of curvature down to ~5 mm. Stretchable, compressible metal–semiconductor–metal photodetectors (MSMPDs) have been fabricated through metal deposition on a wrinkle path [32].

#### 2.1.2. Wave/Wrinkle Structure Patterning

Generally, the wrinkle/wave structures naturally generated in bilayer structures cannot be created in a pattern with a specific direction or shape, due to random properties. However, various applications (e.g., microfluidic system, the direction of nerve cell growth, etc.) of wave/wrinkle structures use controlled patterns.

Pre–patterning [34,69,70], stress distribution control [35,36,48,71], mold patterning [37,38,39], and molecular control [41,49,72] are methods for controlling the random wrinkle structure in the bilayer structure, for creating a specific pattern, including a one–directional wrinkle, herringbones [48], mazes, dots, radial (radially concentric, spoked radial) [66], and hierarchical wrinkles [73].

In particular, pre–patterning is a wrinkle control method that applies controlled buckling due to differences in bending stiffness (Figure 2d). Generally, a patterned mask is attached to an elastic substrate and is exposed to UVO irradiation and oxygen plasma treatment; the exposed region has a relatively higher bending stiffness. On the contrary, the unexposed area is constrained by the difference in bending stiffness in the direction perpendicular to the boundary and has lower bending stiffness; this generates compressive stress in the direction perpendicular to the unexposed region and forms wrinkles in the direction parallel to the boundary during the shrinkage process to the original volume of the elastic substrate. During shrinkage to the bilayer’s original volume, buckling in the high bending stiffness region expands to the low bending stiffness region, relieving the compressive stress and forming a wrinkle perpendicular to the boundary [34,69,70]. The wrinkles are difficult to precisely align compared to other wrinkle generation methods; however, pre–patterning has the characteristic of being able to produce more complex patterns. By controlling the stress distribution during the wrinkle formation, patterned wrinkles can be formed on the bilayer surface. Methods for controlling wrinkling through stress distribution include forming a relief structure on an elastic substrate (Figure 2e) [35] and controlling the direction and sequence of pre–strain (Figure 2f) [36,48,71]. When using pre–strain, the wrinkle direction is determined by the distribution of compressive stresses in the rigid layer that occur upon release, while the wrinkle wavelength proceeds in a larger stress direction. For example, in the case of a bilayer system, which is uniaxially tensioned in the x–axis, a compressive stress can be induced in the x–axis upon release and an x–axis progressive wrinkle pattern is generated. In the case of biaxial tension in the x and y axes, patterns such as herringbones can be produced by adjusting the order and force of the release axes (Figure 2f) [36,40]. Wrinkles, which can be produced by controlling the stress distribution through deformation, have a simple production process and low cost, while the wrinkle pattern is limited. However, several ordered and complex wrinkle patterns can be produced by controlling the stress distribution in the desired direction, by applying a relief structure to an elastic substrate (Figure 2e) [35].

Mold patterning is a wrinkle generation method that applies physical self–assembly, by mechanical stress using a patterned mold; its contour, attached over the bilayer system, allows controlling the mechanical stress and generate an ordered wrinkle pattern [37]. These wrinkles are affected by the period of the line–and–space pattern of the mold and the adhesive force between the mold and the bilayer. If the difference between the intrinsic wavelength of the buckling and the period of the line–and–space pattern on the mold is small, a sinusoidal wave appears (e.g., mold line–and–space: 2 µm, intrinsic wavelength: 2.6 µm). If the difference is large, various non–sinusoidal waves appear (Figure 2g, left) (e.g., mold line–and space: 6.3 µm, intrinsic wavelength: 3.4 µm) [38]. Furthermore, depending on the adhesion of the mold, the convex and the concave wrinkles can be adjusted (Figure 2g, right) [39]. This mold patterning can generate sophisticated wrinkle structures and various patterns. However, mold patterning has a high cost for creating a short period pattern mold, and because the rigid layer and the mold are in direct contact, the possibility of damage to the rigid layer during the removal process of the mold exists (Figure 2g, right).

Molecular control–patterned wrinkles can be generated using the control of stress distribution, the photo–softening effect by molecular isomerization [40,41], and the elastic anisotropy induced through the control of molecular orientation alignment of the bilayer materials [72]. Molecular isomerization alters the Young’s modulus of pre–generated wrinkles through a photo–softening effect and changes in stress distribution. This effect is a phenomenon in which the modulus changes when light of a specific wavelength is applied to isomerization property materials; the modulus varies depending on the ratio of the stable trans–state to the metastable cis state [41]. In addition, rapidly reversible trans–cis isomerization generates forces, which change the stress distribution [40]. Changes in Young’s modulus and stress distribution of the bilayer in random wrinkles can be patterned by deleting the previously created wrinkles or aligning them (Figure 2h). Characteristics of a liquid crystal polymer can be aligned during molecular orientation. The orientation of molecules causes elastic anisotropy, with different elasticities depending on the direction [72]. Elastic anisotropy generates directionally varied compressive stresses during the expansion and shrinkage of a bilayer system and ordered wrinkles. Molecular direction control is used only for materials that can switch their direction by a specific method (e.g., rubbing and photoalignment). Moreover, sophisticated wrinkle structure control and various patternings are possible in a small specific area.

A wrinkle/wave structure in bilayer systems can be used in various fields, such as soft electronics, nano/microstructure fabrication, and biology, by using the adjustability of the maximum stretchability and compressibility of the rigid layer, structural characteristics, component material properties of the wrinkle structure, and various patterning possibilities (Figure 2i). Some applications include dry adhesives [71], capacitors [74], stretchable and compressible circuits [75], cell culture platforms [42], crack–based sensors [76], diodes [35], MESFETs [51] (metal semiconductor field effect transistor), photodetectors [77], pressure sensor [65], and QR CODEs [49].

### 2.2. Stretchable Interconnect

A stretchable interconnect is an interconnect for connecting rigid components in equipment that requires stretchability [78,79,80,81,82,83]. Rigid and bulky components can be connected with no change in electrical conductivity, even with high deformation, due to the design structure or pattern. Research is underway to change the pattern or structure of the interconnect, so that the electrical properties do not change when the device is stretched or bent. The interconnect printed on the substrate is designed in a pattern, such as serpentine or fractal, or the structure is changed to an arc, spiral, or helix. In this way, it is possible to increase the device’s stretchability and maintain stable performance in the case of deformation.

#### 2.2.1. Pattern Design

Interconnects break due to the tensile strength or bending of the equipment. To prevent such damage, research is underway to fabricate interconnects printed on the substrate in various patterns that have been designed so that the interconnect is twisted, rotated, and buckled during tension [78]. Due to this deformation, the interconnect can have a stretchability that does not break, even in tension. Currently, stretchable interconnects are being manufactured using patterns such as serpentine and fractal designs [7,14,84,85,86,87,88,89,90,91,92,93,94,95,96,97,98,99,100,101,102,103,104,105,106,107,108]. The most representative pattern in stretchable interconnects is the former [84,94,102,103,104,105,106,107,108], in which two semicircles are repeatedly connected in a straight line.

**Figure 3:**
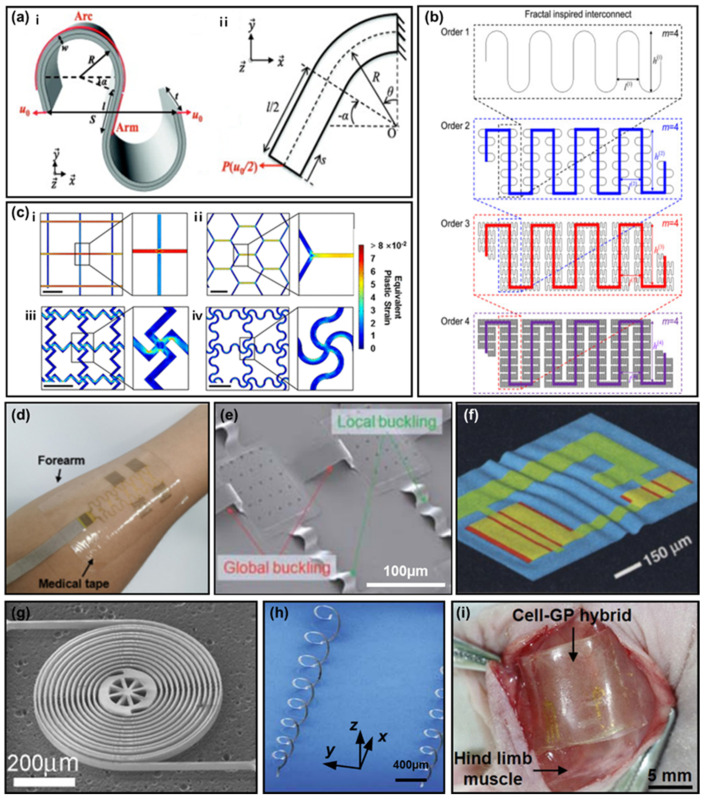
(**a**–**d**) Pattern designed interconnect: (**a**) serpentine pattern [94], (**b**) self–similar pattern [86], (**c**) mesh pattern [89], (**d**) equipment using serpentine pattern interconnect [96]; (**e**–**i**) 3D structural designed interconnect, (**e**) arc [109], (**f**) wave [13], (**g**) spiral [110], (**h**) helix structures [111], and (**i**) equipment using wave structure interconnect [112].

Changes in parameters, such as radius of curvature (R), width (w), angle (α), and arm length (l), to the shape shown in Figure 3a, cause lowering of the stress applied during tension. Through theoretical analysis and simulation, it is possible to check the influence of various variables on the serpentine structure. Interconnects with small w/R, large l/R, and large α are flexible and stretchable, with a few rare exceptions. In addition, stretchability can be greatly improved as the I/R approaches infinity. In addition to the theoretical analysis, FEA was used to confirm the deformation of the interconnect due to strain in the top, front, right, and three–dimensional (3D) view [108]. Based on this, it was confirmed that the serpentine pattern rotates and buckles when tensioned, thereby reducing the stress applied to the interconnect. The stress applied to a serpentine pattern interconnect differs depending on the presence or absence of encapsulation [84]; buckling occurred, and large deformation (26%) was possible when it was not performed. However, buckling did not occur; hence, even a small deformation (9%) received a large stress. Therefore, it can be seen that the serpentine pattern interconnect is capable of greater deformation when buckling occurs. Therefore, the interconnect with a serpentine pattern is a stretchable interconnect that is easy to use, as confirmed by previous studies.

A self–similar structure is a pattern inspired by fractals, as it shows a resemblance to the whole including itself when a part is enlarged [85,86,87,88]. Research is underway to increase the stretchability, by increasing the fractal order [107,108]. Figure 3b is a schematic diagram of fractal–inspired interconnect geometry [86]. This study shows that, as the fractal order of the interconnect is increased from 1 to 4, the stretchability can be improved by ~200 times. Through this, the self–similar structure has greater stretchability compared to the existing serpentine structure. In addition to the serpentine structure, various patterns can be selected and utilized for the self–similar structure. In one study, a self–similar structure was designed using Koch, Peano, Hilbert, Moore, Vicsek, and Greek crosses [88]. The results illustrated the diversity of possibilities with the finite element method and an experimental demonstration. Therefore, by studying the basic patterns of self–similar structures, stretchable interconnects with higher performance can be developed. 

A mesh refers to a pattern in which a shape is constantly connected [89]. The interconnect can be designed with a regular mesh pattern, to improve electrical and optical performance [7,89,90,91,92,93,95]. Figure 3c shows meshes of square, hexagonal, zigzag, and serpentine design; the distribution of plastic strain with respect to tension was simulated for these structures [89]; thus, confirming that the zigzag and serpentine meshes were capable of greater deformation compared to the square and hexagonal meshes. In addition, studies on meshes of various patterns, such as triangular, honeycomb, and kagome, have been conducted [92], confirming that interconnects with a mesh pattern can be practically used in stretchable electronic systems. 

Interconnects with improved stretchability through pattern design are being used in various fields, such as wearable devices and biomonitoring [14,96,97,98,99,100,101]. The device shown in Figure 3d has improved stretchability, with a serpentine pattern interconnect [96]. This device was attached to the skin and utilized for human monitoring. Moreover, a study in which electrophysiological data were collected by attaching to the skin through an interconnect of a self–similar serpentine pattern was confirmed [100]. In addition, stretchable interconnect was used for heart monitoring [14,101]. A serpentine pattern interconnect was used to connect various components, from actuators for electrical, thermal, and optical stimulation, in a moving heart to sense pH, temperature, and mechanical deformation. In this way, the signal from the sensor does not change when the heart moves. This method will be used in more diverse fields if the performance can be improved through additional research.

#### 2.2.2. 3D Structural Design

In addition to the method of designing the pattern printed on the substrate, research on stretchable interconnects that improve stretchability by utilizing various 3D–shaped structures is being conducted [13,32,109,110,111,112,113,114,115,116,117,118,119,120,121,122,123,124,125,126,127,128,129,130,131]. By designing the geometry of the interconnect, the stress applied through spatial deformation during tension is reduced. Accordingly, there is little or no change in the electrical performance of the interconnect, even if there is a deformation in the device. The studies conducted so far show that fractures due to deformation can be prevented using arc, spiral, and helix structures.

The former represents a 3D structure interconnect [121,122,123,128,129,130]; the components are connected by interconnects bent vertically from the substrate [129]. This was manufactured based mainly on the principle of buckling the interconnect by stretching the substrate in advance and then releasing it again. Furthermore, arc structure interconnects fabricated in this way can be theoretically analyzed regarding several variables [129]. The arc structure can have various shapes, depending on the design. As shown in Figure 3e, the shape of the interconnect can be changed by adjusting the buckling [109]. In addition, studies have been conducted to create a new arc structure by utilizing a concave structure between the components or buckling the interconnect in a serpentine pattern, rather than a straight arc [122,130]. Therefore, a more stretchable interconnect can be manufactured by designing patterns and structures at the same time.

In a wave structure, the surface of the substrate and the interconnect appear together in a wavy shape [13,113,116,119]. As shown in Figure 3f, after the substrate is pre–stretched and relaxed, the interconnect and substrate surface together generate a surface wave [13]. During tension, the substrate and interconnect are stretched simultaneously, and the electrical performance is not affected up to the pre–strained length. However, if the length of the pre–strain is exceeded, fracture occurs and the electrical performance is affected [119]. A wave structure can be produced without pre–strain through a photolithography process [116]; it has no pre–strain stress, and the size and direction of the structure can be adjusted. Therefore, wave structure interconnect performance can be further improved through changes in the manufacturing process.

The stretchability of the device can be improved using a spiral structure interconnect [110,118,120,121,131], which is shown in Figure 3g [110]. A spiral structure was applied to achieve high stretchability in monolithic monocrystalline silicon with excellent mechanical and electrical properties. Measured stretches were as high as ~1000% for single helices, while domain extensions were as high as 30 times in the array. Furthermore, it has been confirmed that the spiral structure is highly reversible and does not break up to 412 cycles [118].

A stretchable interconnect was designed with a 3D helix structure [111,114,115,117,125], whose shape is shown in Figure 3h. Currently, helix structures are generally manufactured using buckling; however, other methods have also been proposed. A two–dimensional (2D) micro/nanostructure can be converted into a helix structure by compressing and buckling a certain pattern [111], as shown in Figure 3h. In addition, depending on the buckling pattern, helixes of various structures, such as single, dual, and nested helixes, can be manufactured. In addition to the method cited above, some studies fabricated the helix structure in other ways. For example, using a screw as a template, a helical–structured board was fabricated, and CuNW was transferred to the board to fabricate an interconnect [125]. Here, it was confirmed that the helix structure interconnects had a high elasticity of 700%, without lowering the electrical resistance.

Devices with improved stretchability through various 3D structural interconnect designs are being used in various fields [112,126,127]. As an example, soft electronics that can be attached to the human body were manufactured using a helix–structure interconnect [127]. The stretchability of this device was improved by the helix structure compared to a 2D serpentine pattern. In addition, it was confirmed that signals from sensors, such as electrocardiogram (ECG), electromyography (EMG), electrooculography (EOG), and electroencephalography (EEG), can be measured in this way. Research on fabricating multifunctional implantable devices with improved stretchability using a wave structure has also been conducted [112]; a device was attached directly to the tissue, as shown in Figure 3i. This monitored cell proliferation and differentiation and acted as an electrical stimulator and an electrophysiological sensor in vivo. Therefore, it can be used in various applications that require stretchability of the stretchable interconnect with a 3D structure.

### 2.3. Origami/Kirigami

The origins of origami and kirigami are in ancient papercraft techniques that involve folding and cutting the substrate and extending the use of materials from papers to a broad range of alternatives. Both structures provide a way to apply flat thin planes into 3D structures for various engineering fields, in which a broad range of materials is needed, such as electronics [132,133,134,135,136], optics [137,138], biomedical sensing [15,135,139], robotics [140,141,142,143], and flexible device [134,135,136,138,144,145] applications. Owing to their characteristics, which can modulate the material simply and easily, origami and kirigami structures have been fabricated with various materials, such as metals [133,144,146,147,148], polymers [144,147,149,150,151,152], graphene [153,154], and hydrogels [142], from the scale of meter to micro/nanometer size.

Origami and kirigami structures are similar, as they divide a single substrate into flexible (crease patterns in the former and linkage patterns in the latter) and rigid parts (thin panel without any deformation); while both structures have significant distinctive characteristics from their manner of fabricating structures, as origami folds the plane of a thin film into smaller 3D structures, and kirigami cuts the film into larger extended planes or into 3D structures.

#### 2.3.1. Origami

Origami structures, which are basically folded structures, consist of rigid panels and crease patterns, where the structure is mostly deformed. As a matter of fact, rigid panels are not deformed under bending, stretching, or twisting stress. Crease patterns are designed to be deformed as they are mathematically foldable and have a lower rigidity than other parts of the panel. The rigid panel, which is a standing or base part of the structure, shows less, or no, deformation under stress.

**Figure 4:**
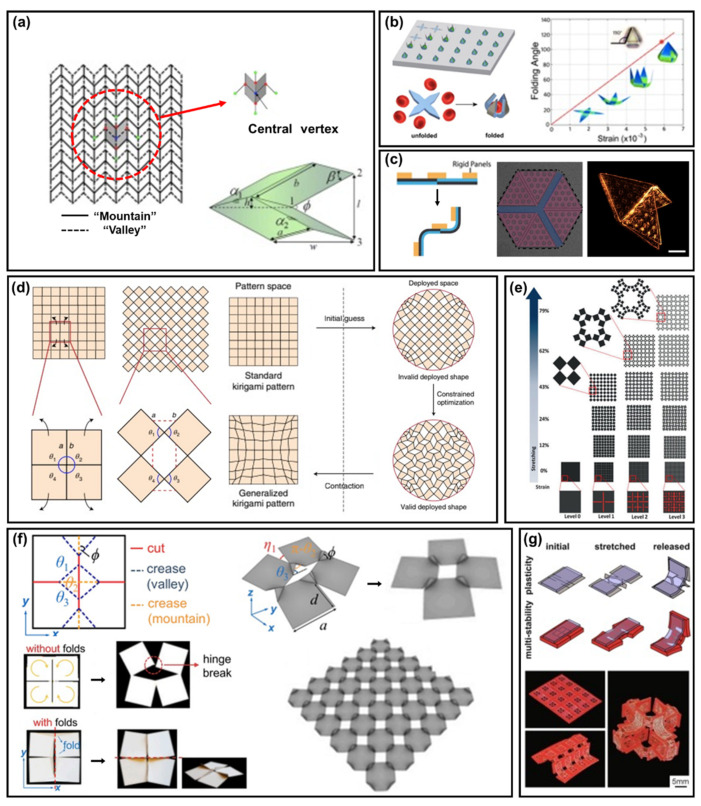
Schematic illustration of: (**a**) Miura origami creases and critical variables [156]. (**b**,**c**) Self–folded origami structures using residual stresses [140,159]. (**d**) Optimized design process for kirigami linkages using an inverse framework method [160]. (**e**) Self–similar concept hierarchical application with enhanced strain [161]. (**f**) Cut and fold structures that combine the advantages of both origami/kirigami structures, and actual images [162]. (**g**) Self–foldable structures using origami and kirigami methods on different multi–layer materials, and actual images [163].

Many crease patterns are used in origami structures; the most common is called the Miura pattern, which has a unique but simple geometry for fabricating structures. This was first designed by Kyoro Miura [155]; all the geometrical properties in Miura origami are determined in the vertex by the lengths (a and b), plane angle (ß), and folding angle (ø), as shown in Figure 4a [156]. The Miura pattern has many mechanical properties, such as tunable Poisson’s ratio, stiffness, and panel directions; thus, being used in various fields, such as flexible electronics [135,136], artificial muscles [157], solar cells [133], and batteries [158], by changing the pattern’s properties as desired.

External forces, such as compression, twisting, and tensile forces, are usually applied by machines or manually as a common way to fold the origami structures, when the scale of the patterns and panels are large enough to fold it. However, as origami methods have many advantages for modulating the material properties with ease, there has been a need to configure the structure and fold it to micro/nanoscales for various applications. The fabrication process of these origami structures mainly utilizes photolithography and etching, due to their high accuracy of patterning; they are sometimes used in 3D printing techniques, with materials such as hydrogel [142] and cellulose [164].

There have been many developments in folding methods for the designing and fabrication process of patterns and structures, and the folding forces used at macroscale in the past cannot be applied to micro/nanoscale structures, due to the scale effect. Thus, origami structures at micro/nanoscale usually use self–folding methods with various stimuli, including capillary force [165], residual stress [140,144,159,163] (Figure 4b,c), light [152], and heat [166,167]. Moreover, there has been research using ‘4D printing’ [168,169] that self–folds with time sequences.

#### 2.3.2. Kirigami

Kirigami structures are basically related to the cutting of a thin substrate into linkages and rigid panels. The linkage pattern is positioned between panels, which changes the deformation depending on its characteristics. Unlike origami, kirigami structures’ deformation behavior changes depending on the composition ratio of panels and linkages; when linkages occupy a higher portion, deformation occurs along linkages and panels under stress. However, when the linkage occupies a smaller portion, the linkage parts are deformed easily, while the panel parts remain rigid. Kirigami structures are more likely to be in 2D and become 3D, depending on the material’s mechanical properties.

Two–dimensional kirigami structures are usually rigid, meaning zero curvature, both before and after the desired stresses; thus, preventing the plane from deforming. The inverse design framework method [160] can be used to design patterns in kirigami, as shown in Figure 4d. This method enables the standard patterns to be changed to more generalized patterns; that is, the desired design of various kinds of deployed states can be calculated by a program that considers important variables, such as length and angle.

Most of the 2D kirigami pattern is designed to be more stretchable than the original substrate material with negative Poisson’s ratios. Among the various kinds of patterns used in kirigami, the self–similar concept is a remarkably effective way of enhancing the stretchability; this design is called hierarchical kirigami [161], as units are hierarchically divided into smaller units with similar morphology, and the same cut patterns are repeated. In hierarchical kirigami design, smaller units start to expand when the larger ones are expanded to their limit. When the units become smaller, from level 0 to 3, the stretchability of the film increases more than 70%, as shown in Figure 4e.

As the kirigami structure depends on the proportion of linkages and panels, 3D kirigami structures can be obtained using the inverse design. In 3D kirigami structures, where the deployed states are obtained in 3D, the linkage design is more critical than in 2D structures. When the panel is flexible enough to be deformed, a 3D kirigami structure can be obtained with simple linkage patterns; however, when the panel is too rigid and stiff to be deformed, linkage patterns enable the structure to be fabricated in 3D, without panel deformation.

Although origami and kirigami structures can be easily used to configure the 3D structures of various materials, there are some problems to overcome. Recently, origami and kirigami structures have been combined to solve the conventional problem, and maximize their advantages, as both methods are simply obtained in thin film. Linkage patterns in kirigami play a vital role; however, they are also vulnerable to being torn off, as they should be thin and narrow for high flexibility. In cut and fold structures [162], the linkage of kirigami is combined with origami crease patterns, as shown in Figure 4f. The combined design enables the expanded angle to be smaller; thus, adding more stability to the structures. Furthermore, with a combined design, the crease pattern can be changed with higher stretchability, while maintaining the pattern’s basic morphology; thus, enabling a larger variety of applications. Unlike combined patterns of origami and kirigami in a single substrate simultaneously, there are many applications of hybrid origami and kirigami structures on different substrates [147,163]. For example, two or more substrates can be assembled for various applications, as shown in Figure 4g, such as self–deploying and self–folding designs, using different materials and stimuli.

### 2.4. Cracks

**Figure 5:**
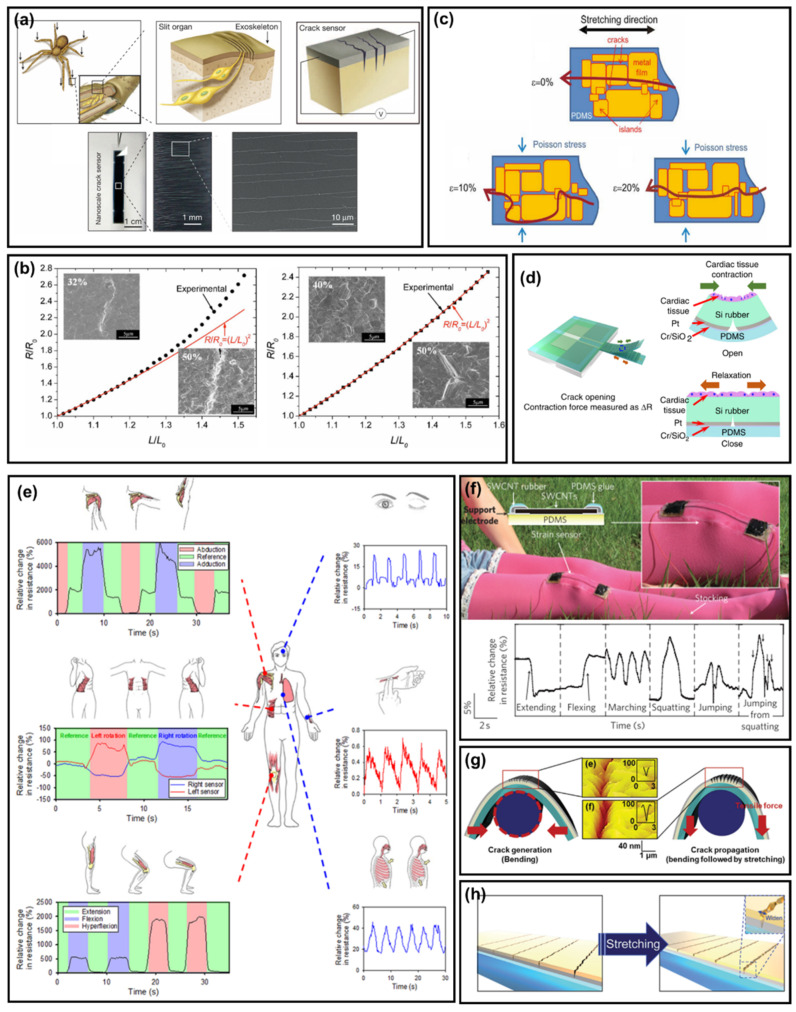
(**a**) Schematic illustrations and images of an ultra–mechanosensitive nanoscale crack junction–based sensor inspired by the spider sensory system [174]. (**b**) Schematic view at 0, 10, and 20% strain of a gold island matrix on PDMS, and corresponding conducting percolation path, after several thousands of stretching cycles [181]. (**c**) Tensile test results with and without a Cr interlayer [182]. (**d**) Concept of a highly durable crack sensor working in culture media [201]. (**e**) Whole–body motion–monitoring system [16]. (**f**) SWCNT–based stretchable wearable devices and relative changes in resistance versus time for breathing, phonation (speech), knee motion, and data glove configurations, respectively [193]. (**g**) Schematic illustrations of crack–based sensor, its mechanism, its geometrical factors for sensitivity, and crack depth–modulated procedure [200]. (**h**) Illustration of the cracks on the sensor before (**left**) and after stretching (**right**). Cracks on the conductive metal layer are induced by those on the interlayer underneath the metal layer [196].

Arthropods have evolved over a long period of time to have a sensitive sensory system [170,171,172,173]. Representatively, the spider slit organ consists of a roughly parallel fissure–shaped sensory lyriform organ for extremely sensitive monitoring of the vibrations of the spider web (Figure 5a) [174]. In recent years, efforts to mimic these organs have changed the perception of cracks, which are now being utilized as another parameter in flexible electronic devices, rather than as material defects due to device failure [175,176,177]. From mimicking external shapes to internal mechanisms, researchers have developed mechanical sensors with high sensitivity, low power consumption, and high reproducibility [175,178,179]. However, there are still many challenges to comprehensively understand the existing sensors [180].

Figure 5b provides a predictive mechanism for stretchable electronics [181]. The resistance increases linearly with the applied strain during the first stretching cycle, as the metal film fractures into locally transverse cracks. Both lateral and transverse cracks open at 10% strain. The contact area between the islands is minimized, which significantly lengthens the conduction penetration path through the network. Thus, the resistance increases. When stretched further, the lateral Poisson compression closes the lateral cracks, which in turn reduces the penetration path, resulting in a decrease in electrical resistance. By controlling the path of electrical penetration through the metal film, the crack can provide a strain function for the strain sensor.

Finite element simulations showed that the substrate delocalizes the strain, so that the metal film can be extended indefinitely, limited only by rupture of the polymer substrate [182]. However, a discrepancy between experiment and theory may arise due to the influence of the very small particle size and inadequate interfacial adhesion [183]. Since debonding plays an important role in this failure mechanism, an adhesive layer that improves adhesion between the film and substrate causes strain localization and cracking of the copper film. Figure 5c shows that, in the presence of a Cr layer, the deviation between the measured resistance and the theoretical prediction of the entire experimental range is reduced. 

Crack–based flexible electrodes can continuously measure cardiac contractility and monitor drug–induced changes in contractility without changing the gage coefficient for up to 26 days (>5 million heart rates) (Figure 5d). Furthermore, when made from stretchable electronic materials, crack sensors can be integrated into clothing or attached directly to the body (Figure 5e); this applies to materials such as Ag [184,185], Au [186], graphene [187,188,189,190], ITO [191,192], and CNT [193,194], allowing for different strain detection ranges and use cases. Wearables and stretchable devices made from thin films of aligned carbon nanotubes break the nanotube films into fissures and islands when stretched, creating bundles that connect the fissures; this mechanism allows the film to act as a strain sensor capable of measuring strains up to 280% (50 times greater than traditional metal strain gages) with high durability, fast response, and low creep (Figure 5f) [193].

In addition, by controlling and optimizing the nanocrack structure, it is endowed with a high linearity and sensitivity and wide operating range [195,196]. It has been confirmed that various human body movements can be detected, including subtle skin deformations, such as joint movements and pulsations [16,197,198,199]. As mentioned earlier, the sensitivity of crack–based sensors can be further improved through control of the crack geometry [180,181,182,183,200,201,202,203,204,205,206,207]. The sensor gage factor generally increases with increasing relative crack depth (Figure 5g) [200]. The depth of the crack can be controlled using a variety of methods, including patterning the existing substrate, applying additional tensile forces after the initial crack is created, and controlling the rate of withdrawal of crack propagation; and it can be easily increased, without changing other geometric parameters (Figure 5h) [196].

### 2.5. Nano/Micro Structured Array

Generally, polymer materials have limitations in Young’s modulus and viscoelastic properties, which prevent the performance of polymer–based sensors from reaching biological sensing systems [208]. Studies that applied nano– to micro–sized structure arrays to polymers have received much attention, to overcome this issue; the structure array provides the sensor with features that depend on its geometric shape. Previous studies have shown that performance is improved by utilizing arrays of nano/microstructures of basic 3D shapes, such as pyramids [209,210], domes [211,212], cones [213], and pillars [17,214,215]. These arrays are commonly applied to polymers such as PDMS [212,216,217,218,219]. In a recent study, a high sensitivity, wide detection range, long–term stability, and fast response time were achieved through basic structure modification and multilayer design. In this section, research using the basic type of nano or microstructure arrays are introduced, as well as the improvements achieved by recent studies.

A unique advantage of integrating nano/microstructure arrays in sensors is to decrease the mechanical Young’s modulus of the system, thereby increasing the change in electrical signal. Common principles for converting mechanical stimuli into electrical signal are piezoelectric [220], piezo–resistive [221], capacitive [216,217,222], and triboelectric techniques [223,224]; all of these detect stimuli due to the polymer deformation of the system. The 3D geometry of a nano/microstructure array achieves the reduction of Young’s modulus by changing the contact area and mechanical strength of the system with various designs. Polymers with nano/microstructure arrays have a smaller contact area with electrodes than planar polymers, due to the three–dimensional geometry of the structure; thus, inducing stress concentration, leading to a greater structure deformation, which in turn causes a change in the conductive path between the electrode and the polymer.

**Figure 6:**
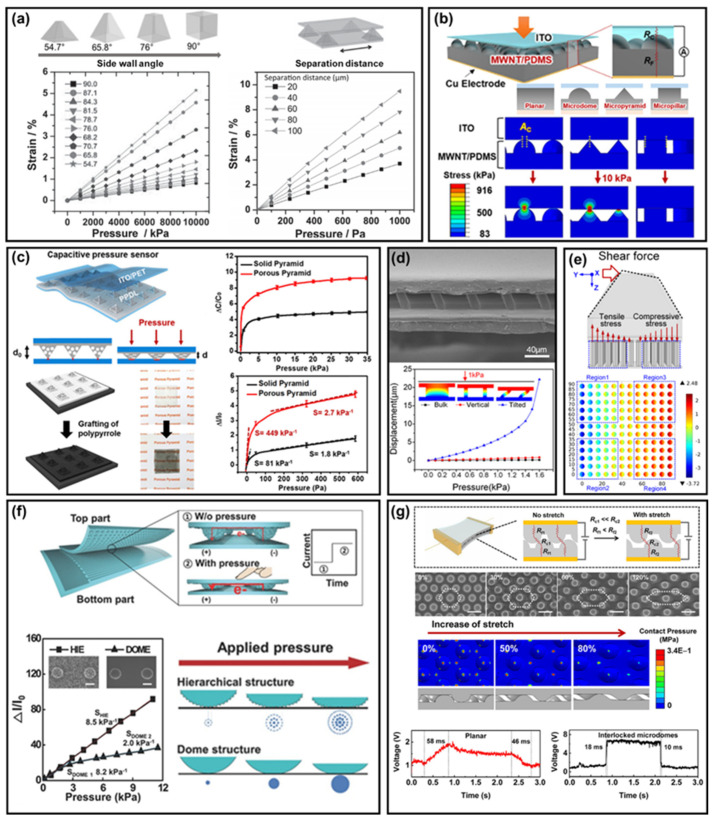
(**a**) Simulation data showing the relationship between shape and strain [216], (**b**) contact area according to nano/microstructure arrays [225]. (**c**) Microarray composed of porous pyramids [227]. (**d**) SEM image of tilted pillar array and finite element method (FEM) simulation [228]. (**e**) Illustration of 3D–axis force sensor and FEM simulation of shear stress [241]. (**f**) Schematic view of sensor operating principle of a hierarchical microstructure array and pressure response [237]. (**g**) Illustration of the interlocking structure sensor operating principle and stretch and sensing response time [211].

Figure 6a shows a simulation of the relationship between strain and pressure caused by a basic nano/microstructure array [216]; Figure 6b shows the change in contact area for each structure in a polymer [225]. Polymers with nano/microstructure arrays have a different pressure sensitivity depending on the structure. The dome structure showed the greatest linear pressure sensitivity among all pressure ranges, which was ~50 times higher than that of planar polymers in a medium pressure range (1–10 kPa).

The structure strength was adjusted using several geometric designs [226]. Figure 6c,d show a porous pyramid and a high aspect ratio structure with lowered mechanical strength through the design of structures constituting a nano/microstructure array [227,228]. The porous structure contributed to the realization of a capacitive sensor with a sensitivity of 44.5 kPa^−1^ and a resistive sensor with a sensitivity up to 449 kPa^−1^ at low pressure, as shown in Figure 6c [227]. High aspect ratio structures have a lower bending strength, using the characteristics of the pillar structure with a high aspect ratio; nano/micropillar arrays have been used for flow rate [215,229], gas [230], pressure [17,231], and tactile sensors [232,233]. Figure 6d provides a nano/microstructure array–based capacitive pressure sensor using a tilted pillar structure [228]; the inclined pillar dielectric layer allows the structure to be more easily bent when pressure is applied to the electrode. Simultaneously, the corresponding deformation mechanism realizes a robust structure, with no air gap between the dielectric layer and the counter electrode interface and high sensor sensitivity. The sensor provided a high pressure sensitivity of 0.42 kPa^−1^ and a small pressure detection of 1 Pa.

Research applying nano/microstructure arrays has advanced into multiaxial force sensing [234,235,236], signal linearity [237,238,239], and response time improvement [211]. Multi–axis force sensing is essential in robotics and many other applications [240]. Sensors with typical pyramidal and dome–shaped nano/microstructures have differences in sensitivity, because the Young’s modulus varies with the strain range, due to the structure’s geometry; this means that the signal exhibits nonlinearity; thus, typical structure nano/microstructure arrays are unsuitable for applications that require linearity. In addition, the nano/microstructure array of the monolayer is still affected by the viscoelasticity of the material, as a mechanism by which the electrical signal change occurs due to the mechanical deformation of the polymer. Thus, viscoelasticity causes a delayed response, which limits the measurable phenomena of sensors composed of a single nano/microstructure array layer.

Figure 6e shows a system for sensing multi–axis forces [241]. A PDMS bump was fixed to an electrode mounted on a micropillar structured array. Depending on the force acting on the bump, the stress applied to the micropillar structure was measured by four piezoelectric elements located on the substrate. The nano/microstructure array using the hierarchical structure partially solved the nonlinear output problem of the sensor. Figure 6f provides a study on the development of a linear pressure sensor utilizing a hierarchical nano/microstructure array [237]; an increase in pressure was converted into an increase in the number of small protrusions in contact; thus, causing the contact area to change almost linearly with pressure. The sensor exhibited a very linear, high sensitivity output of 8.5 kPa^−1^ over a pressure range of 0–12 kPa. An interlocking structure, which was constructed with two microstructure arrays facing each other, was reported as one way to overcome viscoelastic properties [225,242,243,244]. In addition, it has been reported that an interlocking structure exhibits higher sensitivity compared to single–layer nano/microstructure arrays, and it is possible to distinguish electrical signals from various mechanical stimuli [245,246]. Figure 6g provides a sensor implemented with an interlocking structure [211]. In the interlocking structure, the deformation of the nano/microstructure array, and the change in distance and contact area between the microstructures of the two layers, affects the electrical signal. In particular, the distance between the nano/microstructures located in different layers depends on the tensile force acting on the system. The mechanism using the distance change between different layers reduces the influence of viscoelastic properties, enabling fast response output and reduced hysteresis. Furthermore, it can solve the problem of temperature–induced signal change, with a higher sensitivity than single–layer nano/microstructure arrays. Moreover, the distance between two layers of structure reveals different paths, depending on the type of force acting on it. Thus, pressure, shear, torsion, and bending signals can be distinguished. Recently, research has been conducted to improve sensor performance, by simulating various hierarchical [247,248] and natural structures [239] and basic structures. In addition, research on a sensor capable of simultaneously measuring a mechanical stimulus and a temperature [249,250] or a magnetic field [251], beyond distinguishing the stimulus, is in progress.

### 2.6. Textile Structure

**Figure 7:**
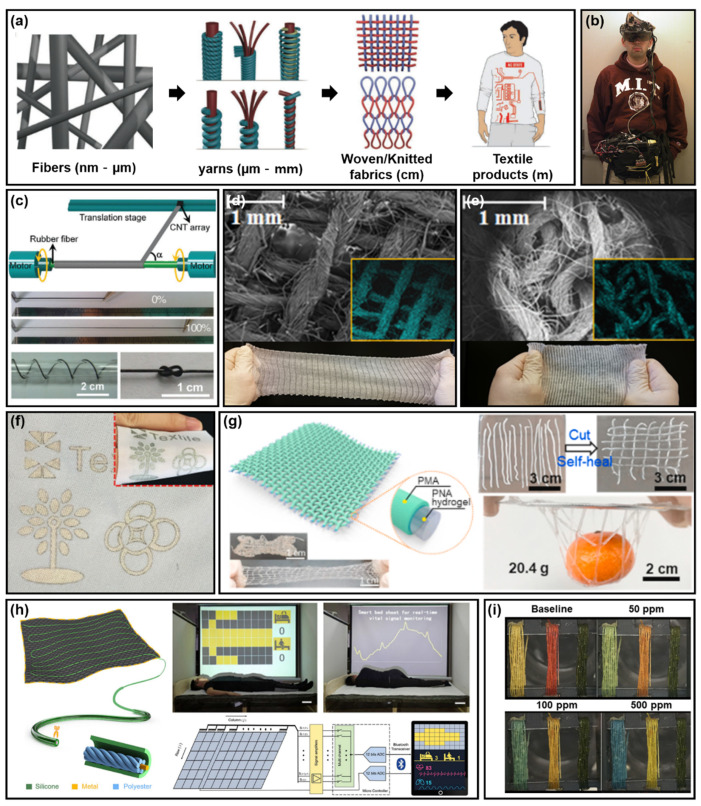
(**a**) Hierarchical structures of textiles at four levels of scale [252], (**b**)in the 1980s, first produced conductive textiles by attaching various type of electronic devices to clothes in the lab of MIT [253]. (**c**) Conductive textile manufactured by winding CNT on a rubber fiber using a motor, durable to various deformations. SEM image of Lyocell–based [255], (**d**) weave, and (**e**) knit coated with a mixture of PEDOT and PPy [256]. (**f**) Textile fabric with pattern deposited using a laser scribing method and electroless plating [257]. (**g**) TENG textile woven with PNA/PMA core sheath fiber and stretching deformation of TENG textile (**left**), photo of the cut PNA hydrogel fiber restored by self–healing and lifting 20.4 g of mandarin oranges using it (**right**) [258]. (**h**) Schematic of a washable functional fiber composed of silicone/metal/polyester (**left**), and figure showing the possibility of real–time sleep monitoring (**right**) [259]. (**i**) Washable gas sensing smart textile showing color change according to ammonia concentration [260].

Textiles, one of the essential elements of human daily life, started with the purpose of protecting the body and has now been developed into an element that enhances individuality and quality of life. Textile can be divided into fiber (nm–µm), yarn (µm–mm), fabrics (cm), and products (m); the durability and elasticity of textile increases from fiber to product [252] (Figure 7a). In the early days of textiles, products with different textures were produced using various materials; however, in the 1980s, textile products using conductive materials were manufactured using conductive materials in the MIT (Massachusetts Institute of Technology) laboratory, making it possible to manufacture clothes with various functions [253]. Conductive textiles use the principle that an external force causes a deformation of the length of the textile, and the electrical resistance of the conductive material changes due to this deformation [254].

#### 2.6.1. Conductive Textile Yarns

Conductive textiles were first produced by attaching various types of electronic devices to clothes in the laboratory of MIT in the 1980s [253] (Figure 7b). In addition, a product with specific functions was manufactured by directly attaching electrical components to the fabric and interconnecting them with the fabric through an arbitrary wiring structure [254,261]. However, this is not practical for daily use, and it is inconvenient, as it restricts the movement of the user. To improve this first attempt, the research team of van Langenhove and T.T Institute proposed a conductive textile using Ag that does not limit the user’s movement; moreover, the wiring is not exposed on the outside of the fabric [262]. Hence, the discomfort of human movement was reduced, and a form of conductive textile developed from this first attempt was exhibited in terms of aesthetics, due to its high similarity to clothing.

Conductive textiles have conductive fibers or yarns that use metal [263,264,265,266,267] or carbon [268,269,270] as fiber–type materials. In addition, conductive polymers such as polyaniline can be used as wiring in textiles [261,262,263,264,265,266,267,268,269,270,271,272,273,274]. The above conductive materials can provide electrical conductivity [275,276], tensile strength [277,278], and mechanical and thermal stability [279] to the conductive textile. For example, aligned multiwall CNT sheets synthesized through chemical vapor deposition were closely wound on elastic rubber fibers in a precisely designed shape. Depending on the angle at which the CNT was wound on the elastic rubber fiber, a fiber with 100% elasticity and a resistance of 0.0886 k ohm/cm was created [255]. Additionally, regarding the production of silver superelastic conductive fibers, T. Ghosh et al. aligned multiwall CNTs attached to highly pre–strained rubbery fibers; thus, confirming that the super–elastic conductive fiber had a maximum elasticity of 1320% and little change in electrical conductivity, even after several thousand cycles of stretching [280]. This study showed that aligned multiwall CNTs can be utilized as conductive fibers and wearable devices, even after thousands of cycles of stretching (Figure 7c).

Afterwards, a fiber–type supercapacitor, in the form of a fiber wrapped with graphene, was developed in the form of a fiber, by twisting two silver FG@3D–G electrodes. Supercapacitors prepared using H_2_SO_4_–PVA gel showed a stable area–to–area capacitance of 1.2–1.7 mF/cm^2^ after bending 500 times [279]. Moreover, a supercapacitor showed 50% and 200% performance for compression and stretching, respectively, while maintaining the electrochemical characteristics; it also showed that the tensile modulus of the fiber increased when using a larger number of electrodes [255].

#### 2.6.2. Weaving and Knitting

Weaving and knitting are methods of processing fibers using manufactured yarns. There are two vertical and separate tread systems in weaving, in which each fiber is closely connected to form a rigid fabric [280,281,282]. In knitting, yarning is a method of manufacturing a fabric with a loop structure, which has the potential to be easily deformed (Figure 7d,e) [256,283,284,285].

Fabrics with woven, knitted construction are made using twist or wrap yarns and have excellent elasticity and durability compared to a single fiber. These exhibit advantages in strain and stress depending on their structure. In the case of knitted fabrics, they have a large elongation value compared to woven fabric, as they have an open and loop–like structure [286,287].

A flexible humidity sensor for human respiration analysis was developed using an open structure, which is an advantage of fabrics with a knitted construction. The flexible humidity sensor made of a fabric coated with graphene oxide showed an ~160% improvement in performance, in terms of hygroscopicity and breathability, which are the limitations of the existing humidity sensors; it was confirmed that this was not affected by the breathing rate [288]. On the contrary, when studying fabricating wearable sensors and heaters using in situ polymerization of pyrrole on knitted cellulose fabric, knitted fabric coated with electrically conductive polypyrrole (PPy) showed an electrical conductivity of 3030 ohm/sq and demonstrated its potential use as a strain sensor, due to its high sensitivity to various operating activities. In addition, it was confirmed that the role of a wearable heater can be performed by using the Joule heating effect after an additional non–wettable treatment [284].

Knitting and weaving have distinct advantages, but also disadvantages, as they show clear limitations. In order to overcome these limitations, a pressure sensor that integrates a woven fabric layer on a knitted fabric layer has been developed. The pressure sensor, manufactured by using a mixture of the two methods, showed a response time of less than 0.4 s in various pressure directions, with a wide sensing range of up to 100 kPa, and a sensitivity up to 0.73 kPa [289] (Xie, Juan et al.). Through this study, the first fabric that integrates knitting and weaving was developed, and it has been applied for the sensing of temperature, pressure, and strain.

#### 2.6.3. Smart Textiles Products

The various advantages of these fabrics have been applied to industry [260,290,291] and energy harvesting [292,293,294,295], and, in particular, have led to the development of smart textiles for healthcare monitoring [256,296,297,298].

A wearable pressure sensor capable of sensing and responding to environmental stimuli was applied to monitor human movement and check health factors, such as pulse. Figure 7f shows the successful implementation of a large–area full fiber–based pressure sensor array on a common fabric substrate. The textile sensor unit achieved a high sensitivity (14.4 kPa^−1^), low detection limit (2 Pa), fast response (≈24 ms), low power consumption (<6 µW), and mechanical stability under harsh deformations. Thanks to these merits, the textile sensor was demonstrated to be able to recognize finger movement, hand gestures, acoustic vibrations, and real–time pulse waves [257].

In other industrial fields, energy harvesting using PNA/PMA composite fibers using dry–wet spinning has been studied. The developed PNA/PMA composite fiber was applied to convert the movement of the human body into electric power. The strain sensing function of PNA/PMA fibers was used to monitor the movement of the human body, and a triboelectric nanogenerator (TENG) fabric woven with PNA/PMA composite fibers was shown to allow remote monitoring, by converting mechanical kinetic energy into electrical power [258] (Figure 7g).

A single–layer soft smart textile for monitoring all physiological parameters during sleep and health management for healthcare fields was studied by Zhihao Zhou et al. A high pressure sensitivity of 10.79 mV/Pa, wide operating frequency bandwidth of 0 to 40 Hz, excellent stability, and washable fabrics enabled dynamic changes in sleeping posture, subtle breathing, and cardio ballistics (BCG); thus, monitoring for the reduction of sleep apnea. This was used in a system capable of diagnosing respiratory syndrome [259] (OSAGS) (Figure 7h).

Last, research on smart textiles that detect harmful gases in industry or daily life is being conducted; it was confirmed that a smart textile, which optically detects dyes reacting to harmful gases, can detect ammonia and hydrogen chloride vapors commonly found in cleaning products, fertilizers, and chemical processes, in the range of 50–1000 ppm. In addition, the difference in sensing performance was ~2% after washing. The possibility of intelligent clothing was confirmed through the manufactured smart textiles [260], which are expected to be applied to multifunctional smart textiles, which are essential in daily life, health monitoring, and industries (Figure 7i).

## 3. Conclusions and Future Perspectives

In this review, we have discussed the different materials that make up the structural design methods. We have provided information regarding soft electronics by summarizing various structural design approaches in parallel, such as wave/wrinkle structure, stretchable interconnect, origami/kirigami, crack, nano/microstructure, and textiles. As a result, various structural design approaches enable interfacing with non–planar surfaces and have shown excellent performance in various fields. Our research is expected to serve as a guide for exploring soft electronics through examples of the latest flexible materials and developed technologies.

Various structure designs have been utilized to fabricate soft electronics. Devices (sensors) having structures such as wave/wrinkle, stretchable interconnect, origami/kirigami, crack, nano/microstructure, and fiber structure were fabricated; and depending on the purpose, high stretchability, repeatability, and sensitivity in performance was provided.

A wave/wrinkle structure is manufactured for the purpose of controlling stretchability and compressibility using a bilayer system and is applied to various applications, such as flexible circuits and biology [42,74]. A wave/wrinkle structure in a polymer surface, it is easy to change the material and shape of the structure; therefore, many applicable properties have been studied [35,51,59,65,73,74]. However, compared to lithography, it has limitations for making elaborate patterns. Therefore, research in various fields, such as flexible devices, biology, and optics, is being conducted, by patterning sophisticated and complex structures in smaller areas and applying them [34,42,43,62,63,65,67,68,69,70,71,75].

Stretchable interconnects are used in various fields, such as wearable devices and bioelectronics [96,97,112,126]. A stretchable interconnect structure is essential in the field of soft electronics, because it has a structure that facilitates the connection of components of the sensor. However, there are limitations, due to the fact that nanoscale patterns and available materials are both limited. Therefore, various studies are being conducted to improve the performance of stretchable interconnects.

Origami/kirigami methods are considered to be good candidates, as their fabrication methods are applicable in micro/nanoscale and the possibility of using various materials that could not to be used due to property limitations. Since origami/kirigami structures are easy to configure, they have been developed and applied in various fields, such as electronics [132,133,134,135,136], optics [137,138], biomedical sensing [15,135,139], robotics [141,142,143], and flexible devices [134,136,138,144,145]. Self–folding methods using lights, heat, capillary force, and residual stresses are being applied to fabricate the structures to be folded in origami structures; however, they still limit the use of materials in their properties. Origami and kirigami structures and their fabrication methods will be developed, as ongoing research is progressing in the field of algorithms with 3D nanoscale structures and cost–effective and precise fabrication methods to fix the problems that have been encountered.

A crack sensor aims to increase the sensitivity and operating range of the sensor through material innovation, and increasing durability through crack control [200,204,205,206,207]. It is easy to manufacture a sensor with high sensitivity and a simple process method. However, as the reproducibility of the sensor is low, research to increase the reproducibility of these sensors is being conducted, using various process methods.

Nano/microstructure arrays are applied to pressure and tactile sensors and are studied for the purpose of increasing the performance of the sensor, such as sensitivity and hysteresis, without changing the material [214,234,235,237,238,239,240]. However, when two or more stimuli act simultaneously, studies on the discrimination of stimuli are insufficient, and additionally, there are limitations due to mass production difficulties and low durability. Therefore, studies on structures having a high durability and mass production using microstructures of various shapes are in progress [299,300].

Textile structures are intended to manufacture a wearable device using a conductive material. There are various shapes, from fibers to fabric products, and they have high stretchability and strength with strains caused by human movement or shape change [274,293,298]. However, they have a limitation, which is their weakness against external contamination and moisture. Therefore, research on a structure capable of maintaining high durability, even in a harsh environment is being conducted [258,260,276].

Structure designs with these various advantages can improve their performance through various studies, to overcome their limitations. Human–machine interface production through improved research is expected to be utilized in the fields of e–skin, wearable devices, and healthcare monitoring.

## Data Availability

Not applicable.
